# Estrogen Impairs Adipose Tissue Expansion and Cardiometabolic Profile in Obese-Diabetic Female Rats

**DOI:** 10.3390/ijms222413573

**Published:** 2021-12-17

**Authors:** Melanie Raquel Martínez-Cignoni, Agustí González-Vicens, Andrea Morán-Costoya, Ana María Proenza, Magdalena Gianotti, Adamo Valle, Isabel Lladó

**Affiliations:** 1 Grup Metabolisme Energètic i Nutrició, Departament de Biologia Fonamental i Ciències de la Salut, Institut Universitari d’Investigació en Ciències de la Salut (IUNICS), Universitat de les Illes Balears, E-07122 Palma, Illes Balears, Spain; melaniemc91@gmail.com (M.R.M.-C.); agustigonzalez.v@gmail.com (A.G.-V.); andrea.moran97@hotmail.com (A.M.-C.); ana.proenza@uib.es (A.M.P.); magdalena.gianotti@uib.es (M.G.); 2 Institut d’Investigació Sanitària Illes Balears (IdISBa), Hospital Universitari Son Espases, E-07120 Palma, Illes Balears, Spain; 3 Centro de Investigación Biomédica en Red Fisiopatología de la Obesidad y Nutrición (CIBERobn, CB06/03/0043), Instituto de Salud Carlos III, E-28029 Madrid, Spain

**Keywords:** diabesity, adipose tissue, tissue remodeling, estrogen, ovariectomy, cardiometabolic alterations, inflammation

## Abstract

It has been reported that 17β-estradiol (E2) can exert beneficial effects against the development of obesity, providing women with a healthier metabolic profile and conferring cardiovascular protection. However, a growing body of evidence questions this role in the context of obesity and diabetes. We focus on the adipose tissue–heart axis to address the question of whether E2 can have metabolically detrimental effects in an obese-diabetic rat model. Female Zucker Diabetic Fatty rats were used: LEAN, fa/+; SHAM, sham-operated fa/fa; OVA, ovariectomized fa/fa, and OVA+E2, ovariectomized and E2 treated fa/fa. The secretory expression profile, tissue expansion parameters and composition of visceral adipose tissue, as well as systemic and cardiac parameters related to insulin resistance, fibrosis, and inflammation were analyzed. Ovariectomy induced an attenuation of both diabetic condition and metabolic dysfunction of adipose tissue and cardiac muscle in fa/fa rats, suggesting that E2, in the context of diabetes and obesity, loses its cardioprotective role and could even contribute to greater metabolic alterations. Adipose tissue from OVA rats showed a healthier hyperplastic expansion pattern, which could help maintain tissue function, increase adiponectin expression, and decrease pro-inflammatory adipokines. These findings should be taken into account when considering hormone replacement therapy for obese-diabetic women.

## 1. Introduction

Increased expansion of adipose tissue in obesity has been related to functional alterations in the tissue such as hypoxia, fibrosis and increased secretion of pro-inflammatory cytokines, which all contribute to a state of low-grade chronic inflammation, increasing the risk of developing insulin resistance and, ultimately, type 2 diabetes mellitus (T2DM) and cardiovascular diseases (CVD) [1,2,3]. In fact, visceral obesity, together with glucose intolerance, dyslipidemia and hypertension, constitute the cluster of disorders known clinically as metabolic syndrome (MetS), which is characterized by an elevated risk of developing heart disease, a stroke and diabetes [4].

Although sex differences in obesity and the associated risk of CVD have been known for many years [1,5,6,7], the mechanism underlying the influence of sex hormones on the development of these pathologies remains poorly understood. The lower prevalence of obesity and diabetes and their associated comorbidities in pre-menopausal women than in age-matched men has been attributed to the protective role of estrogens [1,8]. In fact, visceral adiposity and CVD increases markedly after the cessation of ovarian function at menopause [9]. Estrogens, particularly 17β-estradiol (E2), have been described to exert anti-obesity effects, providing women with a healthier metabolic profile and conferring cardiovascular protection [1,8]. Estrogens have been reported to have multiple actions including improvement in insulin sensitivity, inflammation and oxidative stress [10]. E2 deficiency has been widely associated to body weight gain, increased adiposity and a shift in adipose tissue distribution, effects that are restored by hormone administration [11,12]. Taken together, the evidence on the cardioprotective role exerted by estrogens during the fertile age of women may explain that, in general, they develop CVD later in life than men [13].

Paradoxically, several studies have shown that this sexual dimorphism in cardioprotection vanishes when diabetes is present [5,14,15]. Indeed, in diabetic pre-menopausal women, a dysregulation of lipoprotein metabolism and increased triglyceridemia have been observed [15,16]. Furthermore, these metabolic changes have been associated with the higher risk of cardiovascular complications and mortality diabetic women exhibit in comparison to diabetic men [5]. Moreover, the presence of T2DM reduces the differences in the prevalence of MetS observed between pre- and post-menopausal women [14]. Therefore, evidence suggests that the protective role of estrogens is absent or even could become detrimental in diabetic pre-menopausal women.

This dual role of estrogens in cardiovascular health depending on the presence of obesity-associated diabetes needs further confirmation. Although a better comprehension of the mechanisms underlying the increased cardiovascular risk in obese and diabetic pre-menopausal women would have a great interest for the development of safer hormonal therapies, little effort has been made to date, and preclinical studies in animal models are scarce. Thus, in this study, our aim was to address the question of whether estrogens can have metabolically detrimental effects in a rat model of diabesity. To shed light on this question, experiments were carried out in obese-diabetic female rats undergoing ovariectomy and estrogen replacement, or not undoing these treatments. The secretory expression profile, tissue expansion parameters and composition of visceral adipose tissue (gonadal depot) were analyzed, as well as systemic and cardiac parameters related to insulin resistance, fibrosis, and inflammation.

## 2. Results

### 2.1. Effects of Ovariectomy and E2 Supplementation on Biometric Parameters and Blood Metabolic Profile

**Figure 1:**
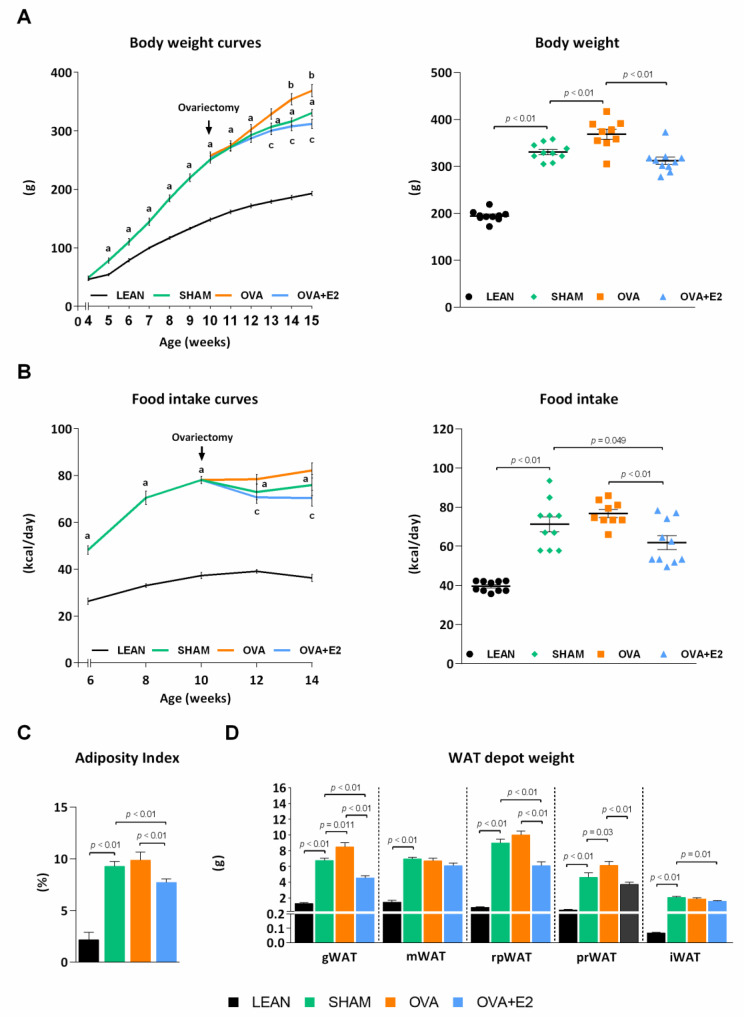
Ovariectomy and E2 treatment effects on body weight, food intake and adiposity in female ZDF rats. (**A**) Body weight and (**B**) food intake over the time (left panels) and at termination of the study (right panels). In A and B the arrow indicates the moment of ovariectomy and the start of E2 or vehicle treatment. (**C**) The Adiposity index corresponds to the sum of all adipose depots weight referred to 100 g of body weight. (**D**) WAT depot weight. WAT, white adipose tissue; gWAT, gonadal WAT; mWAT, mesenteric WAT; rpWAT, retroperitoneal WAT; prWAT, perirenal WAT; iWAT, inguinal WAT; E2, 17β-estradiol. Values are expressed as the mean ± SEM. Differences between obese-diabetic (SHAM) and control (LEAN) animals were analyzed by Student’s *t*-test. Differences between obese-diabetic groups with different hormonal manipulation were analyzed by One-way ANOVA with Fisher’s LSD post-hoc test. Student’s *t*-test: a, SHAM vs. LEAN. One-way ANOVA: b, OVA or OVA + E2 vs. SHAM; c, OVA + E2 vs. OVA.

**Figure 2:**
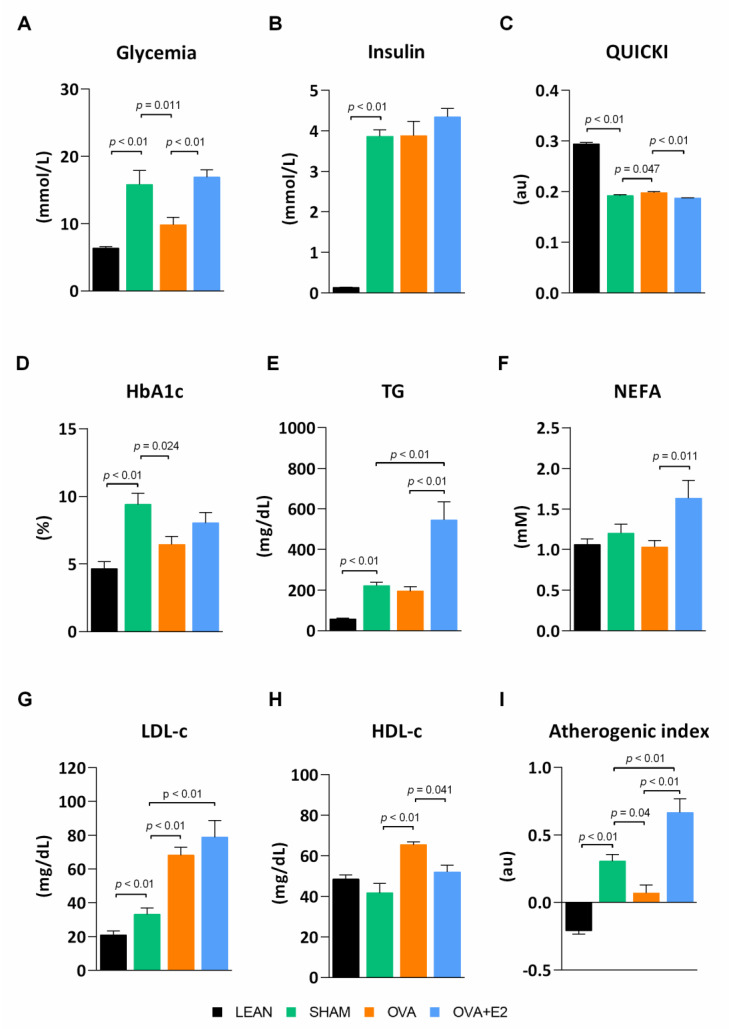
Ovariectomy and E2 treatment effects on serum metabolic parameters. (**A**) Glycemia. (**B**) Insulin levels. (**C**) QUICKI. (**D**) HbA1c. (**E**) TG. (**F**) NEFA. (**G**) LDL-c. (H) HDL-c. (**I**) Atherogenic index of plasma. QUICKI, Quantitative insulin sensitivity check; HbA1c, glycated hemoglobin; TG, triglycerides; NEFA, non-esterified fatty acids; LDL-c, low-density lipoprotein cholesterol; HDL-c, high-density lipoprotein cholesterol. LEAN, control rats (fa/+); SHAM, obese-diabetic (fa/fa) rats undergoing sham surgery; OVA, ovariectomized obese-diabetic (fa/fa) rats; OVA + E2, OVA rats treated with E2 (3 µg/day) for 5 weeks. Values are expressed as the mean ± SEM. Differences between obese-diabetic (SHAM) and control (LEAN) animals were analyzed by Student’s *t*-test. Differences between obese-diabetic groups with different hormonal manipulation were analyzed by One-way ANOVA with Fisher’s LSD post-hoc test.

**Figure 3:**
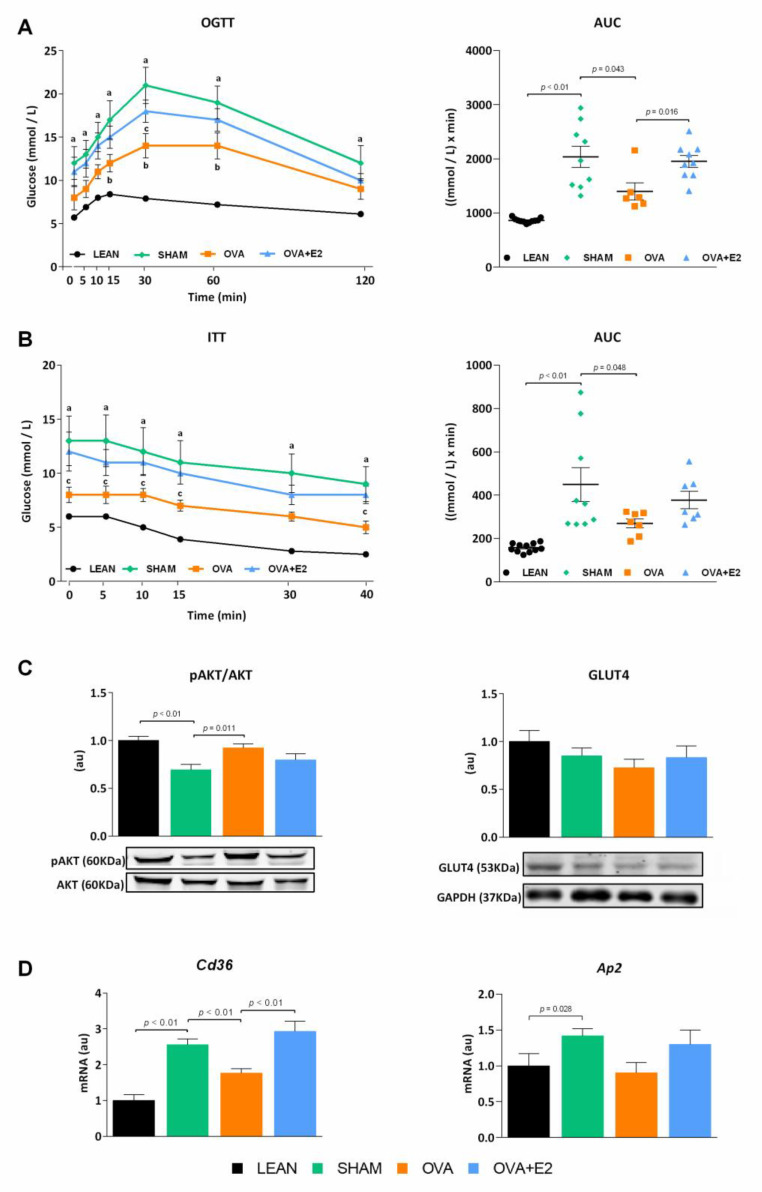
Ovariectomy and E2 treatment effects on oral glucose and insulin tolerance tests in female ZDF rats. (**A**) Oral Glucose Tolerance Test (OGTT). (**B**) Insulin Tolerant Test (ITT). In A and B, left panels represent blood glucose curves and right panels represent AUC. Blood samples were obtained from the tail vein at indicated time points. (**C**) Ratio of pAKT/AKT and protein levels of GLUT4 in WAT. (**D**) *Cd36* and *Ap2* mRNA expression levels in WAT. AUC, area under the curve; AKT, protein kinase B; GAPDH: glyceraldehyde-3-phosphate dehydrogenase; GLUT4, glucose transporter type 4; *Cd36*, cluster of differentiation 36; *Ap2*, adaptor protein 2. In C pAKT protein bands were normalized to total AKT protein intensity, and GLUT4 protein bands were normalized to GAPDH loading control. mRNA levels were normalized to housekeeping *Gapdh* expression. In C and D, data are expressed in arbitrary units (au) related to LEAN group. Values are expressed as the mean ± SEM. Differences between obese-diabetic (SHAM) and control (LEAN) animals were analyzed by Student’s *t*-test. Differences between obese-diabetic groups with different hormonal manipulation were analyzed by One-way ANOVA with Fisher’s LSD post-hoc test. Student’s *t*-test: a, SHAM vs. LEAN. One-way ANOVA: b, OVA or OVA + E2 vs. SHAM; c, OVA + E2 vs. OVA.

ZDF rats are a well-known animal model of MetS widely used to study obesity, diabetes, and associated comorbidities. Unlike fa/fa male rats, females require a high-fat diet to develop overt hyperglycemia [17]. As expected, SHAM (fa/fa) rats showed higher body weight gain and energy intake compared to their LEAN (fat/+) counterparts (Figure 1A,B). In accordance with the higher body weight, SHAM group showed a sharp increase in adiposity with an increment in the size of white adipose tissue (WAT) depots—changes ranged from 5- to 11-fold depending on the depot—(Figure 1C,D). As shown in Figure 2, fa/fa rats reproduce the hyperglycemia, hyperinsulinemia, and high levels of glycosylated hemoglobin (HbA1c) characteristic of patients with T2DM. The diabetic state of fa/fa rats was also confirmed by oral glucose tolerance test (OGTT) and insulin tolerance test (ITT), showing a higher insulin resistance compared to their LEAN counterparts (Figure 3A,B). Regarding lipemic profile (Figure 2), SHAM rats showed higher levels of triglycerides (TG) and LDL-cholesterol (LDL-c) than LEAN rats, supporting the higher atherogenic index of plasma in diabetic animals (values > 0.24).

In order to assess the effects of estrogen deprivation in the context of obesity and diabetes, fa/fa rats were subjected to bilateral ovariectomy. Ovary removal further increased energy intake and body weight gain compared to SHAM group, although the increase in adiposity was only statistically significant in gonadal and perirenal fat depots (Figure 1). Interestingly, despite the higher body weight, energy intake and adipose tissue size, ovariectomy attenuated diabetic condition as shown by lower fasting glycemia and HbA1c levels compared to SHAM rats (Figure 2). This observation was further supported by an improvement in glucose tolerance and insulin sensitivity after ovariectomy (Figure 3A,B). Although no significant differences were found in insulinemia among fa/fa groups, an improvement in insulin sensitivity was suggested by QUICKI index in OVA rats (Figure 2). In terms of serum lipids, ovariectomy had no effects on TG levels in obese-diabetic rats, whereas both LDL-c and HDL-cholesterol (HDL-c) levels were increased. This increase in HDL-c in OVA rats, with no changes in TG levels, accounted for the improvement of the atherogenic index of plasma in this group (value −0.3–0.1).

As shown in Figure 1A,B, E2 supplementation prevented the increase in body weight gain and energy intake induced by ovariectomy. Interestingly, this effect of E2 was also fat depot-dependent, showing a more pronounced decrease in gonadal, retroperitoneal and perirenal depots (Figure 1D). Moreover, E2 treatment reverted ovariectomy-induced changes on glycemia (Figure 2A) and glucose and insulin tolerances (Figure 3A,B). E2 supplementation also reverted the effects of ovariectomy on non-esterified fatty acids (NEFA) and HDL-c levels, and increased TG levels by more than two-fold (Figure 2). As a result, atherogenic index of plasma, which correlates TG and HDL-c levels with risk of atherogenesis [18], greatly increases with E2 supplementation. It is worth mentioning that the marked increase in NEFA levels in E2 supplemented rats could account for a higher influx of NEFA to other organs, promoting lipotoxicity and ectopic fat deposition.

### 2.2. Effects of Ovariectomy and E2 Supplementation on WAT Insulin Pathway and Lipid Transporters

In agreement with the aforementioned improvement of glucose homeostasis and insulin sensitivity in OVA rats, levels of protein kinase B (AKT) activation were higher in WAT of these animals compared to E2-replete (SHAM and OVA + E2) groups. However, no differences were observed in glucose transporter type 4 (GLUT4) protein levels (Figure 3C).

Adipose tissue cluster of differentiation 36 (CD36) and adaptor protein 2 (AP2) are associated to fatty acid accumulation and play a role in inflammation and insulin resistance [19,20]. Expression of these proteins was increased in diabesity conditions, with ovariectomy alleviating this increase. E2-supplementation increased the levels of both *Cd36* and *Ap2* (Figure 3D).

### 2.3. Effects of Ovariectomy and E2 Supplementation on WAT Remodeling

**Figure 4:**
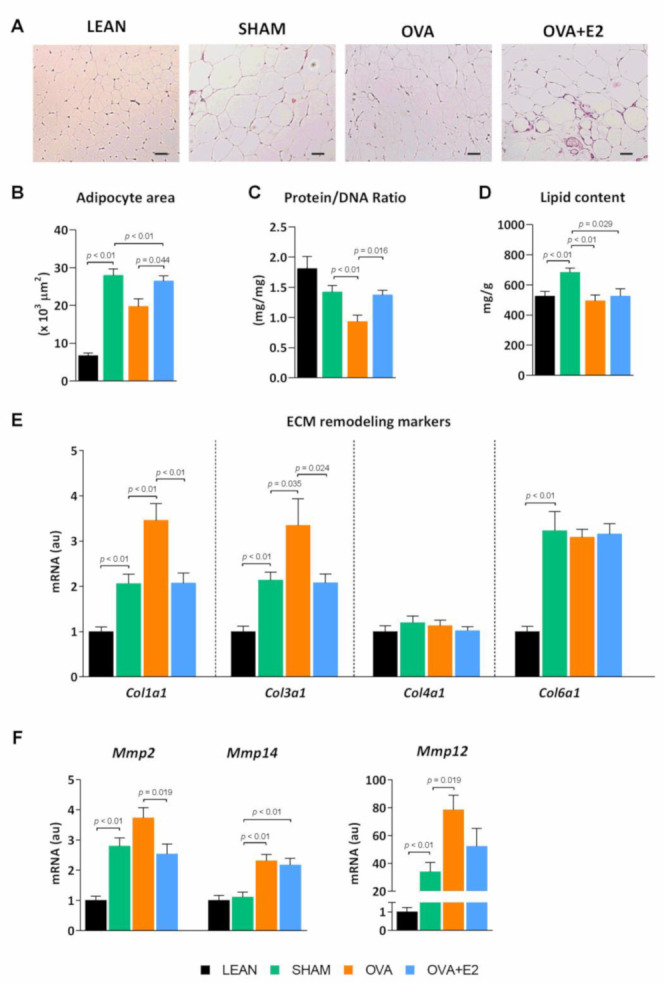
Ovariectomy and E2 treatment effects on WAT composition and expandability in female ZDF rats. (**A**) Adipose tissue histology. Representative slides of WAT hematoxylin-eosin stained sections (scale bar, 100 μm). (**B**) Adipocyte area. (**C**) Protein/DNA ratio and (**D**) lipid content. (**E**,**F**) mRNA expression of ECM remodeling markers. ECM, extracellular matrix; *Col*, collagen; *Mmp*, matrix metalloproteinase. mRNA levels were normalized to housekeeping *Gapdh* expression. In E and F, data are expressed in arbitrary units (au) related to LEAN group. Values are expressed as the mean ± SEM. Differences between obese-diabetic (SHAM) and control (LEAN) animals were analyzed by Student’s *t*-test. Differences between obese-diabetic groups with different hormonal manipulation were analyzed by One-way ANOVA with Fisher’s LSD post-hoc test.

Obese adipose tissue is characterized by dynamic changes in cellular composition and function, which may be referred to as “adipose tissue remodeling” [21]. Hypertrophy is associated to higher hypoxia and inflammation accompanied by the release of pro-inflammatory cytokines and chemokines that facilitate macrophage infiltration, whereas hyperplasia leads to smaller adipocytes with higher insulin sensitivity, and thus, a healthier pattern of fat expandability [22]. In SHAM rats, WAT expansion occurred by means of both hyperplasia and hypertrophy as denoted by the increase in adipocyte area and tissue composition compared to LEAN rats (Figure 4A–D). In comparison, OVA rats showed a lower adipocyte area, as well as lower protein and lipid content, despite the size of the depot being even greater than that of SHAM rats. This suggest that, under ovariectomy conditions, the process of hyperplasia acquires a greater relevance in the expansion of the tissue.

The adipose tissue expansion of fa/fa rats was accompanied by higher expression levels of collagen fiber types Iα (*Col1a1*) and IIIα (*Col3a1*), as well as matrix metalloproteases (*Mmp*) *2*, *Mmp12*, and *Mmp14* (Figure 4E,F). Collagen is the main extracellular matrix (ECM) component and contributes considerably to cell adhesion, migration, differentiation, morphogenesis, and wound healing in the adipose tissue [23]. Metalloproteases are a family of cleaving proteins with pleiotropic effects that participate, amongst others, in vascular remodeling and angiogenesis in adipose tissue [23]. In WAT of OVA rats, *Col1a1*, *Col3a1* and *Mmp12* and *Mmp14* expression was increased compared to SHAM, suggesting a higher degree of tissue remodeling. Interestingly, E2 replacement was able to revert the effects of ovariectomy on adipocyte area, protein/DNA ratio, and *Col1a1*, *Col3a1*and MPP-2 mRNA expression. Thus, E2 restoration induced a more hypertrophic type of adipose tissue expansion.

### 2.4. Effects of Ovariectomy and E2 Supplementation on Expression Levels of Adipokines and Markers of Apoptosis in WAT

**Figure 5:**
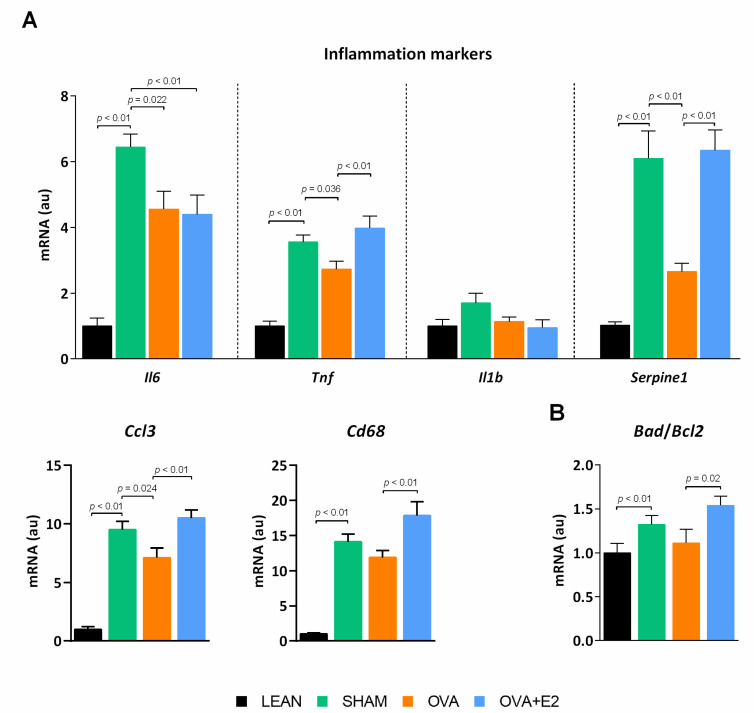
Ovariectomy and E2 treatment effects on (**A**) inflammatory adipokine expression profile and (**B**) markers of apoptosis in WAT of female ZDF rats. *Il*, interleukin; *Tnf*, tumor necrosis factor alpha; *Serpine1*, plasminogen activator inhibitor-1; *Ccl3*, macrophage inflammatory protein-1 alpha; *Cd68*, cluster of differentiation 68; *Bad*, Bcl-2-associated death promoter; *Bcl2*, B-cell lymphoma 2. mRNA levels were normalized to housekeeping *Gapdh* mRNA expression. Values are expressed as the mean ± SEM in arbitrary units (au) related to LEAN group. Differences between obese-diabetic (SHAM) and control (LEAN) animals were analyzed by Student’s *t*-test. Differences between obese-diabetic groups with different hormonal manipulation were analyzed by One-way ANOVA with Fisher’s LSD post-hoc test.

In MetS, adipose tissue contributes to subclinical chronic inflammation by releasing adipokines and cytokines that impair tissue function, as well as that of distant organs and tissues [24]. In agreement, our obese-diabetic SHAM rats showed a higher expression of pro-inflammatory cytokines such as interleukin-6 (*Il*6) and tumor necrosis factor alpha (*Tnf*), and prothrombotic adipokine plasminogen activator inhibitor-1 (*Serpine1*) (Figure 5A) compared to LEAN group. In addition, markers of macrophage infiltration macrophage inflammatory protein-1 alpha (*Ccl3*) and cluster of differentiation 68 (*Cd68*)—as well as apoptosis—Bcl-2-associated death promoter (*Bad*)/ B-cell lymphoma 2 (*Bcl2*) ratio—were increased in SHAM compared to LEAN rats (Figure 5A,B). Ovariectomy decreased the expression of the pro-inflammatory cytokines *Il6*, *Tnf*, *Serpine1* and *Ccl3*, indicating a less detrimental state in adipose tissue of obese-diabetic OVA rats. On the contrary, E2 supplementation abrogated the main effects of ovariectomy on these markers.

### 2.5. Effects of Ovariectomy and E2 Supplementation on the Expression of PPARG and Elements of Adiponectin Signaling Pathway in WAT

**Figure 6:**
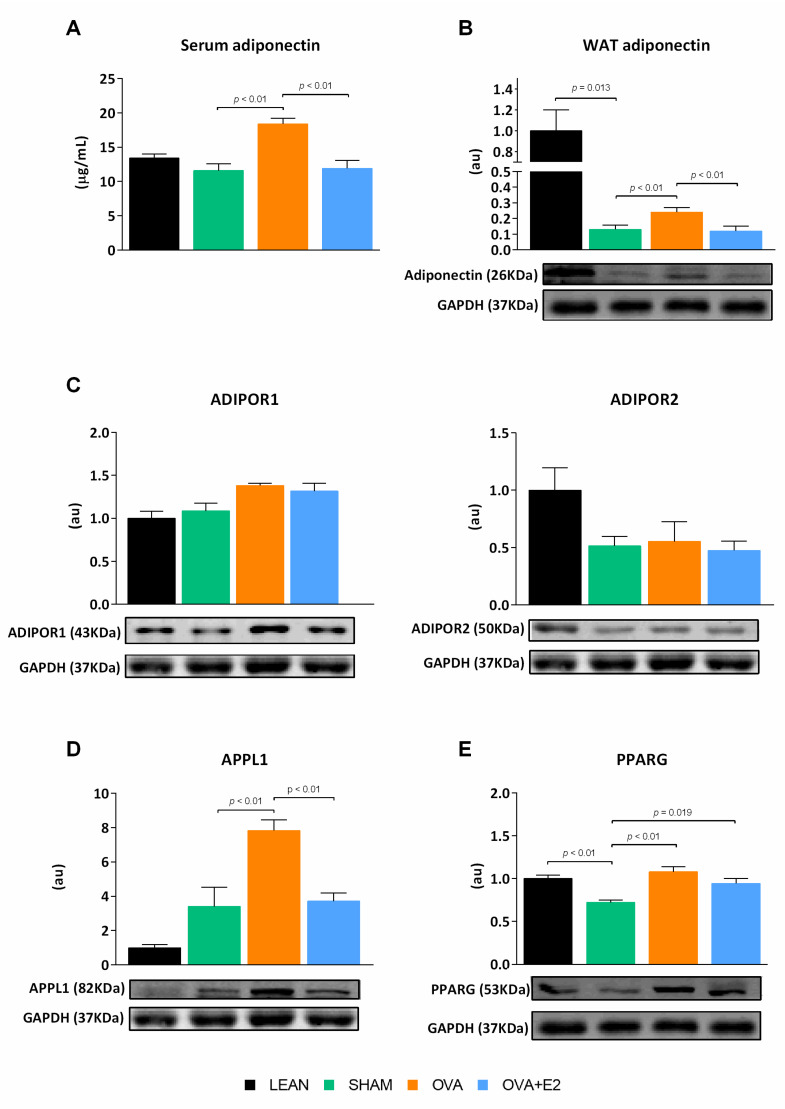
Ovariectomy and E2 treatment effects on adiponectin and mediators of adiponectin signaling pathway in female ZDF rats. (**A**) Serum adiponectin levels. WAT protein levels of (**B**) adiponectin, (**C**) ADIPOR1 and ADIPOR2, (**D**) APPL1, (**E**) PPARG. ADIPOR, adiponectin receptor protein; APPL1, adaptor protein, phosphotyrosine interacting with PH domain and leucine zipper 1; PPARG, peroxisome proliferator activated receptor gamma. Protein bands were normalized to loading control GAPDH protein intensity and expressed in arbitrary units (au) related to LEAN group. Values are expressed as the mean ± SEM. Differences between obese-diabetic (SHAM) and control (LEAN) animals were analyzed by Student’s *t*-test. Differences between obese-diabetic groups with different hormonal manipulation were analyzed by One-way ANOVA with Fisher’s LSD post-hoc test.

Adiponectin has been associated with enhancing insulin sensitivity and glucose homeostasis. As shown in Figure 6A, ovariectomy induced a marked increase in adiponectin circulating levels, which was reverted by E2 supplementation. Consistently, the adiponectin levels in WAT of OVA rats were higher compared to E2-replete groups; despite that, all fa/fa groups showed a marked decrease compared to LEAN rats.

Adiponectin expression is under the transcriptional control of peroxisome proliferator-activated receptor gamma (PPARG), which in turn regulates a large array of key processes in the adipocyte such as mitochondrial biogenesis and β-oxidation [25,26]. In SHAM rats, the reduced PPARG protein levels correlated with the lower adipose tissue adiponectin content compared to their LEAN counterparts (Figure 6B,E). In relation to adiponectin signaling, the levels of the adiponectin receptor (ADIPOR) 2 were reduced in fa/fa rats but not affected by hormonal manipulation (Figure 6C). Conversely, ovariectomy increased PPARG and adiponectin levels, as well as mRNA expression of adaptor protein, phosphotyrosine interacting with PH domain and leucine zipper 1 (APPL1), a component of adiponectin signaling pathway. E2 treatment did not cause a reduction of PPARG, but decreased tissue adiponectin levels as well as APPL1 expression.

### 2.6. Effects of Ovariectomy and E2 Supplementation on Cardiac Insulin Pathway and Fibrosis

**Figure 7:**
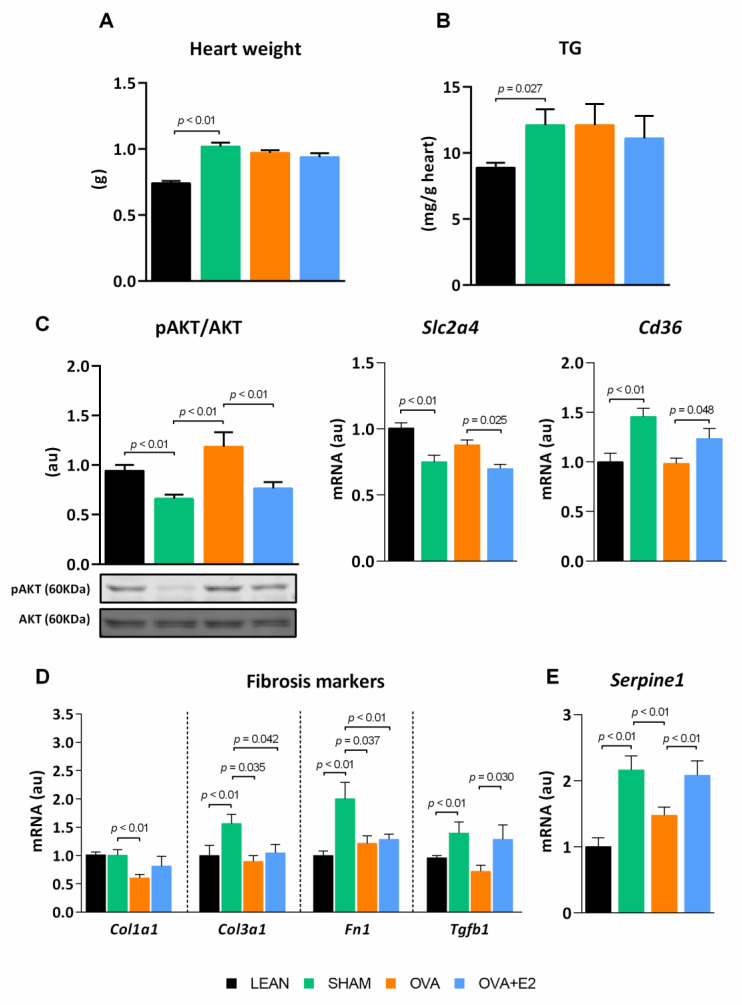
Ovariectomy and E2 treatment effects on cardiac fat content, metabolic and fibrosis markers. (**A**) Heart weight and (**B**) TG content. (**C**) Insulin pathway and expression of *Cd36*. mRNA expression of (**D**) fibrosis markers and (**E**) *Serpine1*. *Fn1*, fibronectin 1; *Tgfb1*, transforming growth factor beta. pAKT protein bands were normalized to total AKT protein intensity. mRNA levels were normalized to housekeeping *Gapdh* expression. Values are expressed as the mean ± SEM. Values in C, D and E are expressed in arbitrary units (au) related to LEAN group. Differences between obese-diabetic (SHAM) and control (LEAN) animals were analyzed by Student’s *t*-test. Differences between obese-diabetic groups with different hormonal manipulation were analyzed by One-way ANOVA with Fisher’s LSD post-hoc test.

Obese-diabetic rats had larger hearts, however, neither ovariectomy nor E2 supplementation changed the tissue weight (Figure 7A). Cardiac tissue of fa/fa rats showed a pattern of AKT activation (Figure 7C) in line with glucose homeostasis and systemic insulin sensitivity, as showed in WAT. Although SHAM rats exhibited a diminished AKT activation in comparison to LEAN group, ovariectomy increased AKT activation, an effect that was reverted by E2 treatment. Notably, AKT activation pattern was mimicked by *Slc2a4*mRNA levels, which confirmed this trend. Both CD36 and GLUT4 play key roles in the heart maintaining fuel flexibility, despite changing physiological conditions, and enabling that, even though the main ATP production is fatty acid oxidation, a fraction of ATP may be obtained by glucose oxidation. Our results showed higher expression levels of *Cd36* translocase in the heart of SHAM rats that were reduced by ovariectomy and restored by E2 treatment. ZDF fa/fa rats exhibited fat accumulation in cardiac muscle, which was not affected by hormonal manipulations (Figure 7B).

Hyperglycemia and insulin resistance have been linked to fibrosis of the heart ECM, a process known to impair cardiac contractibility. In comparison to LEAN, obese-diabetic SHAM rats showed a higher expression of fibrotic components of the ECM such as *Col3a1* and fibronectin (FN) 1, as well as a higher expression of major inductors of fibrosis such as transforming growth factor beta 1 (TFG-β1) and *Serpine1* (Figure 7D,E). Ovariectomy reduced the expression of *Col1a1* and *Col3a1*, *Fn1*, TFG-β1 and *Serpine1*. On the contrary, E2 treatment reverted the effects of ovariectomy on TFG-β1 and *Serpine1* expression, whereas *Col1a1*, *Col3a1* and *Fn1* expression remained unaltered.

## 3. Discussion

Although most of the studies carried out in general population and in normal weight and obese animal models indicate that estrogens could play a protective role against cardiovascular risk, there is a growing body of evidence questioning such a protective role in a context of diabesity. The literature suggests that E2 could have a dual role depending on the pathophysiological situation [1,5,16,27]. In fact, the deterioration of glucose homeostasis increases the risk of cardiometabolic diseases in pre-menopausal women [1]. Specifically, T2DM confers a higher relative risk of cardiovascular events in women compared to men of the same age [5,27,28]. In this work, we found that in a situation in which obesity and diabetes converge, ovariectomy improves glycemic and lipemic profiles along with a healthier expansion of visceral adipose tissue and cardiometabolic profile.

As expected, our female fa/fa rats on a high-fat diet developed an early obesity that led to the onset of insulin resistance and a fully diabetic phenotype, showing impaired glucose and lipid serum levels. In our model, obese-diabetic OVA rats showed a less harmful metabolic profile, with lower glycemia and dyslipidemia, as well as higher glucose tolerance and lower insulin resistance in comparison with E2 replete (SHAM or OVA + E2) groups. This better preservation of insulin sensitivity in ovariectomized fa/fa rats was further supported by higher AKT activation levels in WAT and heart. Notably, estrogen supplementation was able to abrogate many of the effects of ovariectomy, such as improved glucose and insulin tolerance, suggesting that E2 plays a negative role in a diabesity context. Although ovariectomy did not modify circulating TG levels in fa/fa rats, their large increase in response to E2 replacement is noteworthy. These effects have also been described in other T2DM models [12,29] and could be attributable to E2 [30]. In agreement, dyslipidemia and hypertriglyceridemia are commonly reported in diabetic pre-menopausal women and post-menopausal women on estrogen replacement therapy, contributing to the higher CVD risk [14,15,16,31]. The impaired lipemic profile shown by our obese-diabetic OVA rats supplemented with E2 agrees with these previous reports and supports E2 contribution to dyslipidemia in a diabesity context.

The results obtained in E2-supplemented diabetic OVA rats contrast with the widely accepted protective role of estrogens. However, most of the studies in ovariectomized animal models showing a detrimental effect of hormone depletion on glycemic and lipemic control have been performed in normoglycemic, non-obese and non-diabetic rats, or diabetic, but non-obese rats [32,33]. The most widely used experimental designs fail to use animals that simultaneously exhibit obesity and overt diabetes. Conversely, we have used a genetic model of diabesity in which the animals show clear alterations of metabolic homeostasis. Although it cannot be ruled out that the absence of functional leptin receptor (Lepr) may influence the effects of ovariectomy and E2, similar results reported in non-genetic rodent models of obesity and diabetes (high-fat diet plus streptozotocin) and obese-diabetic humans suggest that Lepr absence is not required for the detrimental effects of E2 in a diabesity context [5,14,15,34]. Moreover, previous studies in ovariectomized fa/fa rats failed to find effects of E2 [17] or even reported protective effects on glucose metabolism [35]. However, many of these studies used standard chow diet and, therefore, animals did not reach overt hyperglycemia [17,35]. It should be noted that female fa/fa rats are required to be on a high fat diet to develop overt diabetes, therefore, these reports cannot be directly compared to the present study.

Adipose tissue is recognized as an endocrine organ capable of releasing signal molecules that target distant organs such as the liver, heart, and blood vessels. Thus, adipokines exert key roles in regulating energy homeostasis, insulin sensitivity or inflammation [2,36,37]. Chronic inflammation in WAT may cause whole-body insulin resistance in obese-diabetic animals [38,39]. In our animal model, diabesity induced a huge increase in the expression of pro-inflammatory (*Il6*, *Tnf* and *Il1b*) and prothrombotic (*Serpine1*) adipokines and chemokines (*Ccl3*) as well as markers of macrophages infiltration in WAT. Given the involvement of PPARG in adipose tissue inflammatory response, its downregulation in WAT of SHAM and its upregulation in OVA rats could be associated with the expression profile of *Tnf* and *Il6*, as well as markers of macrophage infiltration, as previously reported by other studies [40]. At the same time, the levels of adiponectin are markedly decreased in our obese-diabetic rats, consistent with previous reports [41,42]. Although the adiponectin levels in the WAT of fa/fa rats were 10 times lower than those in LEAN rats, ovariectomy caused an almost twofold increase in adiponectin expression in the WAT of obese-diabetic rats. Considering the anti-inflammatory and insulin-sensitizing actions of adiponectin [43], the increased expression of this adipokine, and its higher circulating levels, could be related to the healthier metabolic profile of OVA rats and the amelioration of diabetes-associated disorders in this experimental group. Consistently, OVA rats presented an activation of adiponectin signaling pathway through ADIPOR1 and APPL1 as suggested by their increased expression levels. The upregulation of APPL1, through its role facilitating the crosstalk between adiponectin and insulin signaling pathways [44,45], and interacting with AKT [45], favors insulin sensitivity. Thus, higher levels of adiponectin in the serum and WAT of OVA rats could explain the improvement in glucose tolerance and insulin sensitivity associated to E2 deprivation, reflected by a greater activation of AKT in WAT and a reduction in glycemia and Hb1Ac, at the systemic level. Results from epidemiological studies suggest the existence of a negative association between E2 and adiponectin circulating levels in both pre-menopausal and post-menopausal women [46,47,48]. Moreover, E2 has been positively associated with insulin resistance, a pathological state suggested to be mediated indirectly through female sex steroids’ effects on adiponectin expression and adiposity [46].

In adipose tissue, CD36 participates in fat utilization and storage, but also, in the onset of inflammation in response to excess fat supply [49], thus contributing to adipose dysfunction [19,49]. *Cd36* was upregulated in the WAT of our obese-diabetic rats, in agreement with previous results obtained in both obese and diabetic patients [50] and obese Zucker rats [51]. Ovariectomy downregulated WAT *Cd36* expression, which could be associated with a lower recruitment of pro-inflammatory macrophages to adipose tissue, ameliorating tissue inflammation and therefore improving both systemic and tissue insulin sensitivity. Similarly, mice fed on a high-fat diet with a deletion of *Cd36* showed an improved insulin action, and a reduction in both macrophage recruitment and inflammatory profile of adipose tissue [19]. The adipocyte lipid chaperone AP2, which in our model follows the same profile reported by CD36, seems to play a more critical role in macrophage lipid accumulation [20] and could also contribute to the healthier profile of obese-diabetic OVA rats.

In our study, all groups of fa/fa rats exhibited similar adiposity index, but significantly different levels of insulin sensitivity, which could explain differences in WAT expansion (healthy vs. pathological). In fact, it has been proposed that dysfunction, rather than an excess of adipose tissue, might be the pathological process underlying the link between obesity and metabolic disease [52]. The expansion of adipose tissue requires remodeling and reorganization of ECM to allow the enlargement of adipocytes (hypertrophy), and adipogenesis (hyperplasia) [53]. ECM remodeling is crucial for healthy adipose tissue expansion [23]. Sex hormones have pronounced effects on adipose tissue mass and distribution [1]. We found clear hormone-dependent differences in the cellularity and remodeling capacity of WAT between fa/fa groups. Adipocyte hypertrophy is closely linked to adipose tissue dysfunction, inflammation, and insulin resistance, while hyperplasia is linked to an improvement of insulin sensibility [22]. In our model, WAT expansion in E2-replete rats was mainly promoted by both hyperplasia and hypertrophy, whereas in obese-diabetic OVA rats, despite having greater depots of visceral fat, the growth of WAT occurred mainly through hyperplasia, which has been related with a healthier tissue growth, playing a protective role in the development of metabolic diseases associated with obesity [52]. In addition, in OVA rats, the accumulation of COLIα and COLIIIα fibers in the ECM of WAT could represent an adaptive response to the uncontrolled increase in the size of the adipose depot, thus avoiding cell hypertrophy [22]. Moreover, the increased expression of *Mmp2*, *Mmp12*, and *Mmp14* in WAT of OVA rats, could additionally contribute to a better regulation of adipogenesis and angiogenesis processes. This hypothesis would agree with the observation that in diabetic patients the lower accumulation of collagen fibers in adipose tissue is associated with higher hypertrophy of adipocytes [54]. Thus, a high amount of collagen fibers seems to limit the growth capacity of the adipocyte, promoting tissue expansion due to hyperplasia [54]. In our study, the changes observed in ECM remodeling in WAT of obese-diabetic OVA rats—greater accumulation of collagen and metalloproteinases—suggest the existence of an adaptive fibrosis. This process could act as limiting to the excessive growth of adipocytes, promoting a healthy adipose expansion, avoiding hypoxia and, ultimately, adipocyte death, as shown by apoptotic markers. On the contrary, the remodeling of the ECM in the WAT of E2 replete groups would represent a maladaptive fibrosis, more permissive with the enlargement of adipocytes. This mechanism would result in a greater degree of hypoxia, adipocyte death and release of inflammatory factors [53,55].

In this study, we also analyzed parameters of insulin sensitivity, fibrosis, and inflammation in the heart, in order to corroborate whether hormonal manipulation and subsequent metabolic changes could have a differential impact on the heart of obese-diabetic animals. In fact, epidemiological studies in general population indicate that diabetes weakens the cardio-protection of pre-menopausal women typically associated with estrogens [5,27]. In agreement with this, our results indicate attenuated, or even detrimental, effects of E2 on the heart of obese-diabetic rats. The reduced cardiac AKT activation and increased HbA1c serum levels, showed by both SHAM and OVA + E2 groups in our study, have been previously associated to diabetic cardiopathy [56]. A recent study linked ovarian hormones and E2 to the exacerbated myocardial dysfunction in female diabetic rats [34] and suggested that E2 could interrupt adiponectin signaling at the cardiac level. As a matter of fact, in our study, serum adiponectin levels in SHAM and OVA + E2 rats were at the same level of LEAN rats but did not translate into a healthy cardiometabolic profile maintenance, which could be due, at least in part, to the interference of E2 with adiponectin signaling.

Our diabesity rat model showed a marked fat deposition in cardiac muscle, similar to other diabetic models [57]. Despite cardiac fat content being similar in hormonal manipulated groups, the expression profile of fatty acid and glucose transporters was altered in myocardium in a E2-dependent manner. The E2-replete groups showed higher expression of *Cd36* and lower *Slc2a4* compared to OVA rats, suggesting that hormonal deprivation could allow greater metabolic flexibility in the heart, essential to meet energy demand [58]. Interestingly, *Cd36* expression, which was downregulated in the myocardium of OVA compared to E2-replete rats, has been linked to inflammation and calcium imbalances altering heart function [59]. Furthermore, OVA rats also showed lower expression of *Col1a1* and *Col3a1* as well as *Tgfb1* in the heart, evincing a lower degree of fibrosis in the myocardium. Collagen fibers and fibronectin in ECM of heart have been associated to cardiac muscle stiffness and diastolic dysfunction [60]. In accordance with the literature, this response could be consistent with increased AKT phosphorylation in OVA rats because cardiac fibrosis, during diabetic cardiomyopathy, has been linked to hyperglycemia and dysregulation of the AKT pathway [56]. In addition, insulin resistance has been associated with increased *Serpine1* and *Tgfb1* expression in the heart [61] contributing to the development of cardiac fibrosis.

In conclusion, in our obese-diabetic model, ovariectomy induced an amelioration of diabetic condition and metabolic dysfunction of adipose tissue and myocardium. Our results support the idea that, in a diabesity context, E2 loses its cardioprotective role and even contributes to greater metabolic alterations. This improvement in diabetes and cardiometabolic profile could be related, in part, to the healthier expansion observed in visceral adipose tissue of ovariectomized animals, which could help maintain tissue function, increasing the expression of adiponectin, with insulin-sensitizing effects, and decreasing that of proinflammatory adipokines, inducers of insulin resistance. These findings provide evidence of unhealthy effects of E2 in T2DM and, therefore, this should be considered when prescribing hormone replacement therapy to diabetic women.

## 4. Materials and Methods

### 4.1. Animal, Diets and Treatments

All animal procedures performed in this study were in accordance with general guidelines approved by Institutional Ethics Committee (nr. 3515/2012) and EU regulations (2010/63/UE). A total of thirty-two Zucker Diabetic Fatty (ZDF-Lepr^fa^/Crl) female rats were bred in our animal facility at University of Balearic Islands by breeding homozygous (fa/fa) males and heterozygous (fa/+) females supplied by Charles River Laboratories (Barcelona, Spain). All animals were housed in a controlled environment (22 °C and 65 ± 3% humidity) under a 12 h light–darkness cycle with free access to food and water. At ten weeks of age, animals were divided into 4 groups (*n* = 8); control ZDF lean non-diabetic rats (fa/+, LEAN); sham-operated ZDF obese-diabetic rats (fa/fa, SHAM); ovariectomized ZDF obese-diabetic rats (fa/fa, OVA) and ovariectomized and E2-treated ZDF obese-diabetic rats (fa/fa, OVA + E2). Bilateral ovariectomy was conducted in sterile conditions and E2 (3 μg/day) was administered via ALZET^®^ osmotic mini pumps (Charles River Laboratories, Barcelona, Spain) located in the interscapular area. Successful ovariectomy was confirmed by uterus atrophy—LEAN 0.40 ± 0.01 g; SHAM 0.36 ± 0.03 g; OVA 0.12 ± 0.01 g; OVA + E2 0.40 ± 0.01 g. Pumps were filled with E2 dissolved in ethanol, DMSO and saline (3:10:7) solution according to the manufacturer’s instructions. LEAN fa/+ rats were fed with a standard diet (3438 kcal/kg; Ref. A04/A04C/R04) (SAFE, Paris, France). All ZDF fa/fa rats were fed with a high-fat diet (4059 kcal/kg; Ref. D12468) (Research Diets, New Brunswick, NJ, USA). The estrous cycle was regularly determined by microscopic examination of fresh vaginal smears. All animals were in the pro-estrous phase at the time of sacrifice. Blood was collected from tail vein before proceeding to the sacrifice, glucose was determined using Accu-Chek Nano device (Roche Diagnosis, Basilea, Switzerland) and HbA1c was assessed using colorimetric assay (CrystalChem, Elk Grove Village, IL, USA). At fifteen weeks of age, after 6 h-fasting period, animals were decapitated without the use of anesthetics to avoid interference with blood analysis. Blood was allowed to clot and was then centrifuged (×g, 10 min, 4 °C) to obtain serum that was stored at −20 °C until analysis.

Gonadal WAT and heart were quickly removed, frozen in liquid nitrogen and stored at −80 °C until analysis. A fresh sample of WAT was used for histological analysis. Mesenteric, retroperitoneal, perirenal and inguinal depots of WAT were also removed and weighted to adiposity index calculation.

### 4.2. Glucose and Insulin Tolerance Tests

OGTT and ITT were performed one week prior to sacrifice after 6 h-fasting period. For OGTT, rats received an oral administration of glucose solution (1 g/kg body weight), and blood glucose levels were measured over a period of 120 min from the vein tail, using Accu-Chek Nano device (Roche, Indianapolis, IN, USA). For ITT, rats received an insulin subcutaneous injection (1 U/kg body weight) and blood glucose levels from the vein tail were measured over a period of 40 min, using Accu-Chek Nano device (Roche, Indianapolis, IN, USA).

### 4.3. Serum Parameters

ELISA kits were used to determine insulin (CrystalChem, Elk Grove Village, IL, USA) and adiponectin (CrystalChem, Elk Grove Village, IL, USA). TG, HDL-c, LDL-c and NEFA levels were measured using colorimetric assays (Cromatest, Barcelona, Spain; Wako Chemicals GmbH, Neuss, Germany). QUICKI index (Quantitative insulin sensitivity check index) was calculated as [1/ [log (fasting insulin (μU/ml)) + log (fasting glucose (mg/mL))]] [62,63]. Atherogenic index of plasma was calculated as log (TG (mM) / HDL-c (mM)) [18].

### 4.4. Tissue Sample Preparation and Determinations

A piece of WAT or heart was homogenized with a polytron disperser (IKA T10 basic ULTRA-TURRAX, Königswinter, Germany) in a proportion 1:10 in STE buffer (250 mM sucrose, 20 mM Tris-HCl, 40 mM KCl, 2 mM EDTA, pH 7.4). Homogenates were stored at −20 °C with protease and phosphatase inhibitors (HALTTM; Thermofisher Scientific, Waltham, MA, USA) until Western blot analysis. Protein concentration was determined by BCA method (Thermofisher Scientific, Waltham, MA, USA). Heart triglycerides were determined in fresh homogenate (STE buffer without proteases and phosphatases inhibitors) with a commercial kit according to manufacturer indications (Cromatest, Barcelona, Spain; Wako Chemicals GmbH, Neuss, Germany). Total lipid content of WAT was quantified by Folch method [64].

### 4.5. Morphological Analysis of WAT

A total of twenty mg of fresh WAT was collected and fixed in 4% paraformaldehyde for 24 h. Next, samples were dehydrated with 75% ethanol for 48 h, paraffin-embedded and sections were cut and stained by hematoxylin-eosin. Histological images were captured in an inverted optical microscope and adipocyte area was calculated with Image J software (https://imagej.nih.gov/ij/, accessed on 15 October 2021).

### 4.6. RNA Isolation and Real-Time PCR

Total RNA was obtained from 0.3 g of WAT or 0.1 g of heart using Tripure^®^ Isolation Reagent (Roche Diagnostics, Basel, Switzerland), following manufacturer’s instructions. A total of one µg of total RNA was reverse transcribed to cDNA using M-MLV commercial kit (Invitrogen,Waltham, MA, USA). The reaction was set up as follows: 25 °C (10 min), 37 °C (50 min), 70 °C (15 min) and 4 °C in a Gene Amp 9700 thermal cycler (Applied Biosystems, Waltham, MA, USA). cDNA solution was diluted 1/10 and stored at −20 °C until was analyzed. Real time PCR was performed using LightCycler^®^ 480 System II (Roche Diagnostics, Basel, Switzerland). A total of 2.5 µL of cDNA dilution was added in 7.5 µL of SYBR master mix containing sense and antisense primers (0.374 µM each; IDT Technologies, Leuven, Belgium) in a final volume of 10 µL. The amplification program consisted of a pre-incubation denaturation step (95 °C, 2 min), followed by 40 cycles reaction (denaturation 95 °C, 5 min; annealing at primer-dependent temperature, 10 s; and extension 72 °C, 12 s). Glyceraldehyde 3-phosphate dehydrogenase (*Gapdh*) was used as housekeeping gene in all expression analyses. Oligonucleotide sequences and product length in real time PCR are detailed in Table 1.

### 4.7. Western Blot Analysis

A total of thirty-five μg of protein from homogenates were fractioned on SDS-PAGE gels and electrotransferred onto a nitrocellulose membrane. Membranes were blocked in a commercial TBS blocking buffer (Odyssey, Li-Cor, NE, USA) for 1 h and incubated overnight with the corresponding primary antibody solution. Antibody for adiponectin (26 kDa, 3553) was supplied by ProSci (Poway, CA, USA); antibodies for ADIPOR1 (43 kDa, sc-99183), ADIPOR2 (50 kDa, sc-46755), GAPDH (37 kDa, sc-365062), GLUT4 (53 kDa, sc-7938) and PPARG (53 kDa, sc-7196) were purchased from Santa Cruz Biotechnology (Dallas, TX, USA); antibodies for AKT (60 kDa, cs-2920), pAKT (60 kDa, cs-4060) and APPL1 (82 kDa, cs-3858) were supplied by Cell Signaling (Danvers, MA, USA). GAPDH was used as protein loading control. Protein bands were detected using Odyssey Infrared Imaging 9120 system (Li-Cor, Lincoln, NE, USA) and analyzed using Image Studio^TM^ Lite software (Li-Cor, NE, USA). Precision Plus Protein Dual Color Standard (Bio-Rad, Hercules, CA, USA) was used as molecular weight marker.

### 4.8. Statistical Analysis

All data are expressed as the mean ± standard error of the mean (SEM) of 8 animals per group. Statistical differences between obese-diabetic (SHAM) and control (LEAN) animals were analyzed by Student’s test. Statistical differences between obese-diabetic groups with different hormonal manipulation were analyzed by One-way ANOVA with Fisher’s LSD post-hoc test. Threshold cycle (Ct) values of real-time PCR were analyzed using Genex software version 6 (MultiD Analyzes AB, Göteborg, Sweden), considering efficiencies of each pair of primers which were calculated experimentally. All statistical analyses were performed using a statistical software package (Graphpad^®^ Prism version 9, San Diego, CA, USA) and a *p* value < 0.05 was considered statistically significant.

## Figures and Tables

**Table 1:** Oligonucleotide primer sequences used in real time PCR amplification and product length.

Gene	Accession Number	Forward Primer (5′->3′)	Reverse Primer (5′->3′)	Product Length (bp)
*Ap2*	XM_039095687.1	CCGATCCACTCCTTACCT	GCCACCGTGACCTTGTAC	254
*Bad*	NM_022698.1	AGAGTTTGAGCCGAGTGAGCACT	CCGGGTCTCCATAGTCC	186
*Bcl2*	NM_016993.1	CTTCTTTGAGTTCGGTGGGGTGGA	GAAATCAAACAGAGGTCGC	151
*Ccl3*	NM_013025.2	TGCCCTTGCTGTTCTTCTCT	AAAGGCTGCTGGTCTCAAAA	152
*Cd36*	NM_031561	CTCACACAACTCAGATACTGCTG	TCCAAACACAGCCAGGACAG	200
*Cd68*	NM_001031638.1	CCCGAACAAAACCAAGGTCC	CTGCGCTGAGAATGTCCACT	196
*Col1a1*	NM_053304.1	GGAGAGAGCATGACCGATGG	GGGACTTCTTGAGGTTGCCA	184
*Col3a1*	NM_032085.1	TCCCCTGGAATCTGTGAATC	TGAGTCGAATTGGGGAGAAT	63
*Col4a1*	NM_001135009.1	CTCTGGGGACAACATCCG	TCTTCTCATGCACACTTGGC	397
*Col6a1*	XM_008767345.2	GGGACACACGTCTTCAGGTT	CCATGACTGATTGTTGTTGGG	150
*Fn1*	NM_019143.2	CAGCCCCTGATTGGAGTC	TGGGTGACACCTGAGTGAAC	73
*Gapdh*	NM_002046	CTGGTGGTCCAGGGGTCTTA	CCACTCCTCCACCTTTGACG	156
*Il1b*	NM_031512.2	CTGTGACTCGTGGGATGATG	GGGATTTTGTCGTTGCTTGT	210
*Il6*	NM_012589.2	TGTTCTCAGGGAGATCTTGG	TCCCAGGTAGAAACGGAACTC	485
*Mmp12*	NM_053963.2	GGCTGCTCCCATGAACGAG	GAGGTGTCCAGTTGCCCAG	177
*Mmp14*	NM_031056.1	AGGCCAATGTTCGGAGGAAG	GTGGCACTCTCCCATACTCG	154
*Mmp2*	NM_031054.2	ATGGTCGGGAATACAGCAGC	AGCTGTTGTAAGAGGTGCCC	195
*Serpine1*	NM_012620.1	TCTCTCCCTATGGCGTGTCC	GGCATCCGCAGTACTGATCT	188
*Slc2a4*	XM_041665189.1	TATTTGGCTTTGTGGCCTTC	CGGCAAATAGAAGGAAGACG	201
*Tgfb1*	NM_021578.2	GCAACAACGCAATCTATGAC	CCTGTATTCCGTCTCCTT	300
*Tnf*	NM_012675.3	CTGAACTTCGGGGTGATCGG	CTTGGTGGTTTGCTACGACG	151

All primers were designed to work under a high annealing temperature of 60 °C.

## Data Availability

The data presented in this study are available on request from the corresponding author.
